# Latham on Diseases of the Heart

**Published:** 1847-07

**Authors:** 


ART. II. *Latham on diseases of the heart. Review continued from page* 30.

The work of Dr. Latham in so far as our analysis extended in the preceding number, treated of Endocarditis and Pericarditis as associated with arthritic rheumatism. The occurrence of these affections disconnected from rheumatism is next considered. But little space is devoted to them in this aspect, for, as we have already quoted, Dr. L., declares that they have very rarely fallen under his observation, except in that connection. He gives some account, first, of endocarditis, which he has observed independently of rheumatism. In these cases various and complicated affections of other organs have co-existed, and the primary deviations from a state of health were not well ascertained. He thinks that endocarditis may not infrequently become developed during the last stages of the progress of other diseases. That it may bear relations to some other forms of disease, as well as to rheumatism, is probable, but clinical observations have not established any definite principles presiding over such relations. The subject requires farther elucidation by means of the collection and analysis of a greater number of cases than are, as yet, aavailablo.

Pericarditis, disconnected from rheumatism, is altogether of more frequent occurrence than endocarditis. Indeed, Dr, L., states that "there is no structure of the body more liable to inflammation than the pericardium," one-half of the bodies of adult subjects, exhibiting on dissection some traces of its existence at some period of life. But in many instances the lesions which declare its existance are trivial, and the disease had been of a trifling character. The white spots so often found on the surface of the heart, are now regarded as the products of slight inflammatory action. Limited as are the portions of the heart's surface affected in these cases, it is highly probable that the morbid action was sufficient to have produced appreciable sounds of attrition; and the affection would be much oftener diagnosticated, if the custom were adopted of examining the heart in all cases, whether symptoms directing attention to this organ are present or absent.

It is evident that in point of danger, there is no comparison between cases of endocarditis and pericarditis in which the morbid action is of little intensity, and restricted to a small space; for, while the latter exposes the patient to but little, if any risk of ulterior evils, the former may induce confirmed valvular lesions, and thus lead to extensive and fatal structural changes in the whole organ

Dr. L. remarks, that in the larger proportion of cases of acute pericarditis, in which the affection has occurred independently of rheumatism, it has been overlooked during life. This has been true of the history of several cases which we have collected, and we presume, that all who have been in the habit of making autopsies, can testify to the same fact. This arises, in part, from the latency of the affection in many cases, as respects the symptoms exclusive of those furnished by physical exploration, and the too common neglect of the latter in cases in which the symptoms do not compel the attention of the patient and practitioner to be directed to the condition of the heart. It is also, in part, explained by the fact that in nearly all instances the pericarditis occurs as a complication of other diseases. The liability to overlook the disease should be borne in mind, and should enforce the importance of never neglecting, in cases of every description, examination of the præcordial region with the hand and ear. Dr. Hope enjoins this in a particular manner, and states that it was his habit to ascertain the condition of the heart in all cases of acute disease, as uniformly as of the pulse, tongue, and other prominent sources of vital symptoms.

With respect to the occurence of pericarditis as a complication of other diseases when not connected with rheumatism, Dr. L., states that in no instance in his own experience has it presented itself as an isolated disease. It is especially apt to be associated with inflammation of similar structures—the peritoneum and pleura. Like endocarditis, but much more frequently, it is often developed shortly before the fatal termination of various acute diseases,occuring just be-fore death,to which it may directly contribute in a considerable degree

Upon an analysis of cases communicated by Corvisart and Andral, the fact of its occurring for the most part as a complication is sustained.

Our author proceeds to treat of the *immediate results of endocarditis and pericarditis.* The consequences of inflammation of the endocardium may be the deposit of lymph in specks, or in layers, over a greater or less extent of surface; its accumulation in masses as large as a pea, or cherry-stone, or larger, which are sometimes pendulous into the cavity of the ventricle; ulceration, which may destroy more or less of a valve, or lay bare the muscular substance, and sometimes penetrate from one auricle to the other. These are destructive forms of injury incompatible with life: there are others less destructive, and capable of slow reparation. The local consequences of Pericarditis are, the effusion of lymph universally, or in patches; agglutination of the pericardium to the heart; effusion of fluid, from a few ounces to four pints, which may be transparent, flaky, purulent, or sanguinolent.

In view of these local effects, in either instance, the importance of striving, not only to cure the inflammation, but to cure it *quickly* is sufficiently obvious. The condition of the system at large has not a little to do with the prospect of ultimate reparation. The prospect is comparitively small in children of weak, scrofulous constitutions, and in adults who are habitually in infirm health. Patients during the restorative efforts of the system sometimes fall into a state of anæmia, which may greatly retard, if it does not prevent recovery.

When the course of the affection is unfavorable, dropsy generally supervenes, commencing with œdema of the feet, which may be at first attributed merely to debility, but gradually it affects the cavities, and distends the cellular texture throughout the body. Recovery, however, occasionally takes place after these most unpromising events have occured.

Coincident with the heart affection various diseases are sometimes observed, such as maniacal delirium, fatuity, epileptic or tetanic convulsions, chorea, &c.

Reparation from these affections of the heart may take place to such an extent that life is safe for the time being, but more or less degree of permanent unsoundness remains. Under these circumstances, the period to which life may be continued is various. "It may be a few months only, or a few years; or it may be many years, even ten, or twenty or thirty."

These cases terminate in two ways:—first, by the effects of a renewal of disease in the unsound organ; second, by the direct consequences of the detriment which the organ has received, without any revived inflammation being involved. Dr. L., considers these two methods at length, *and, first as regards renewed, or secondary inflammations.*

A person who has been affected with inflammation of the structures of the heart, which has left the organ in a permanently damaged state, is constantly liable to a new accession of inflammation, not only in connection with recurrence of rheumatism, (if this disease have been experienced) but from "exposure to cold, intemperate indulgences, or some unusual bodily effort." Now under these circumstances the diagnosis is less easy and sure, than when the heart was previously healthy. The physical signs, which are our chief reliance in previous cases, now fail to afford such precise information. We quote the author's words on this subject:—

"In the first inflammation of the pericardium, there is the exocardial murmur made by the moving of its roughened surfaces upon each other. But in after inflammation of the pericardium, exocardial mumur there is none, and none can there be if its surfaces adhere completely. And if they adhere partially and there be a murmur, it will not have the proper attrition in it, and so will want the exocardial character. In the first inflammation of the endocardium, there is the endocardial murmur, made by the recent lymph deposited upon a valve; and the murmur continues ever afterwards, when the valve so far falls short of perfect reparation as to remain thickened or puckered. And then in after inflammations, observe the puzzle. There is the permanent murmur of the old unsoundness and the recent murmur of the new disease; but how much is due to the old, and how much is to the new, is too delicate an affair for the nicest ear to discriminate."

The signs denoting the presence of renewed inflammation are not only equivocal and uncertain in cases of lesser intensity, but even in the severer and fatal instances. For its recognition we are to take into consideration its frequent coincidents and accompaniments. There are attacks of acute rheumatism, pneumonitis, pleuritis, or fever. Experience declares that "when any one of these befals a man whose heart has been left unsound by a prior inflammation, then inflammation is apt to be renewed in it afresh."

The author adds,—"when under such circumstances, and in such a subject, the heart which habitually palpitates, and is habitually uneasy, suffers an increase of palpitation and of pain, then its inflammation should be assumed as a fact." "I believe," he continues, that whenever the heart is re-inflamed by a fresh attack of rheumatism, there is almost always a a tremendous accession of palpitation and pain. Oftentimes, however, when the palpitation and pain have been the greatest, they have been most easily subdued, for these are no sure measure of the severity of the disease, and no sure warning of its fatal result."

The following are the authors's remarks on the treatment of renewed inflammation:—

"As to the kind of medical treatment, I would remark generally, first, with respect to bleeding, that if you now direct this mode of depletion with the view of *entirely* stilling the violent action of the heart and arteries, you propose a false and impossible indication of practice; false, because this violent action is in part permanent, and has not to do with the present conditions of disease: impossible, because no quantity of bleeding short of that which would kill the patient would be adequate to the purpose; and, secondly, with respect to mercury, that all which can now be done is common ly within the reach of other remedies, and therefore that *commonly* it is unnecessary.

"Leeches applied to the region of the heart, will, by the immediate effect which they produce, test the sort of inflammation you have to deal with, and show whether any and what other remedy will be needed in counteraction of it. If they at once afford marked relief, they thus denote both that the inflammation is easily controllable, and that they, without the aid of any other remedy properly antiphlogistic, will be able to control it. And so it will turn out in the majority of cases. But if they afford no marked relief at once, or, still more, after their repeated application, then they plainly proclaim the inflammation beyond their power to cope with, and they call for the help of mercury (as at first) to withhold it from a fatal issue; but this does not often happen.

"In the treatment of these secondary inflammations, it must always be borne in mind that they *are* secondary. We must restrict our practice to the purpose of removing so much of the disease as is superadded by the present attack, and abstain from pushing either bleeding or mercury to such an extent as we should if we proposed to play a successful after-game for the complete cure of the disease of the heart, which is impossible.

"But these secondary attacks of inflammation which people suffer and recover from, and suffer and recover from again and again, do they always add something to the permanent unsoundness of the heart? I cannot tell; but probably not always."

The effect of medical treatment in these cases of secondary inflammation, may be to relieve the morbid action so that the condition of the patient is no worse than before, or, on the other hand, notwithstanding our therapeutical efforts, the permanent unsoundness of the organ is more or less increased. The latter will be manifest by an increase of the palpitation, præcordial uneasiness, dyspnœa, &c., symptoms which had preceded the new recurrence of inflammatory action, but in a lesser degree. The patient's susceptibility to future repetitions of inflammatory attacks is also greatly increased.

"When secondary inflammation has been thus a few times renewed in the heart, and the patient, though his life be saved, has reverted after each attack to a worse condition than before, it is remarkable how little it takes to light it up afresh. A rheumatic fever is sure to do it; even a common febrile catarrh may do it; nay, it will sometimes appear to light itself up spontaneously; and thus with a cause, or without a cause, it will return, or seem to return, at short intervals of months or weeks, and the patient perhaps will at last die of an attack much less severe than many a one that has preceded it."

We come now to the second class of consequences of endocarditis and pericarditis, viz:—those arising from the damaged state in which the heart is left, without involving any renewal of inflammatory action. The unrepaired structure of the organ becomes almost necessarily the element of further material changes in it. This set of consequences is the more frequent. Renewal of inflammation happens to but few, but "farther disorganization of the heart growing out of the elementary unsoundness left by inflammation, certainly happens to the vast majority and, if accurate measure could be taken during life, would probably be found to happen to almost all." Dr. L., distinguishes the latter train of consequences as "*unsoundness* from *disorganization*," while the unsoundness attributable to the immediate effects of inflammation he calls "*unsoundness* from *disease*." The former are the direct, the latter the remote, or ulterior results of the inflammatory condition.

The following is his description of the permanent lesions found on dissection.

"In looking over such records as I possess, of dissections made where death had taken place at various periods of many months or of many years after the attack which did the original detriment to the endocardium, I find that the morbid appearances may be reduced to a few, to opacity and thickening of the membrane, to marks of perfect and imperfect cicatrisation, and to breach of surface or solution of continuity.

"The opacity and thickening, vary much in their extent. Sometimes they are confined to a single valve or to part of one only, to its free edge; sometimes they affect more than one, generally two, the mitral and the aortic valves; and they occasionally extend to the valves of both sides of the heart, and pervade both their proper membranous expansions and the tendinous cords proceeding from them. Further, this same opacity and thickening belong sometimes to other portions of the endocardium besides those which form the valves and their appurtenances, especially to the lining of the left ventricle nearest the aorta and to the whole lining of the left auricle.

"Besides such general opacity and thickening a particular valve sometimes presents a hard elevated line or ridge where it is especially thickened, or a small spot where it is indented or depressed, looking like a complete cicatrisation in one case and an incomplete cicatrisation in the other.

"Sometimes a valve is perforated or cribriform, or it wants a portion at its edge, or a tendinous cord is snapt in two, and its ends are hanging loose within the cavity of the ventricle.

All these lesions lead to farther disorganization by the same mode, viz:—by occasioning *more or less impediment in the passage of the blood through the heart.* Their existence is demonstrated during life, by the presence of the bellows, or endocardial murmur in some of its modifications. But, it is to be noted, that the loudness of this murmur is no evidence of a correspondingly great amount of structural disease. On the contrary, Dr. L., states, that, as a general fact, the louder the murmur, the less the impediment. The physical explanation of this fact will be obvious on a little reflection.

There is no uniformity in cases as to the rapidity with which ulterior unsoundness, or unsoundness from disorganization, progresses. In some instances in which the presence of the physical sign reveals the fact of existing lesions, the patient is wholly unconscious of any disturbance in the organ; and perhaps, very little, if any disturbance is produced, the lesions being such as to occasion the morbid sound, without any notable impediment in the passage of the blood. The general remark that the bellow's murmur does not invariably denote changes of structure which are of a fatal, or even serious character, is important to be borne in mind. In other cases, impediment co-exists with the murmur, but not being considerable in amount, the patient only experiences palpitation and dyspnœa after exertion, or upon excitement of the circulation from any cause. Under these circumstances the morbid condition may continue for years with slight, if any augmentation. In cases wherein the lesions are of a graver character, more or less increased force of the heart's action, or palpitation, is constant, which is, of course, aggravated on any exertion. Nevertheless, under these circumstances comfortable health for many years is not an inpossibility,*

Without considering, at present, the various results to which progressive unsoundness of the heart gives rise, the important fact which clinical experience declares is this:—"The evil consequences to life and health arising out of the heart's permanent unsoundness left by endo-carditis are often either stationary at a small amount for years, or very slow to advance or accumulate."

The foregoing remarks have related to unsoundness incident to endocarditis. That resulting from pericarditis presents this difference as respects diagnosis;—we have not the continuance of the characteristic physical sign. The exocardial or attrition murmur always ceases with the active disease, and does not remain to denote the permanent disorganization which is left. Hence, it often happens that the results of pericarditis are disclosed after death, when nothing of the kind had been suspected during life. The effect of pericarditis, certainly in the vast majority of cases, if not invariably, is more or less adhesion and obliteration of the pericardial cavity. This may be partial or complete; it may take

*For a case illustrating these remarks, see report of editor's clinique in another article of this number.

place apparently by agglutination, without any evidences remaining of an intervening solid deposit, or the amount of organized lymph which connects the membrane to the surface of the heart may be half an inch in thickness, and so of every intermediate degree. Adhesion is doubtless a conservative process in these cases, for, although it rarely ensures a completely healthy condition of the organ, it secures a condition of greater safety than if a cavity remained to be filled with serum, or to become the seat of purulent matter. The imperfect accomplishment of adhesion is sometimes a serious evil, the heart presenting after death what appears to be a series of abscesses, occasioned by purulent deposit in the spaces free from adhesion. Dr. L. entertains the opinion that obliteration of the pericardial cavity is not incompatible with perfect health. This (if we recollect aright) was not the opinion of Hope. The latter observer found, that in all cases it led inevitably to hypertrophy, with or without dilitation. That it does so in the vast majority of instances is also the opinion of Dr. Latham. It may be a question whether acute pericarditis may ever occur without leading to more or less adhesions, but the fact that it may occur and recovery take place so complete that not the least evidences of cardiac trouble or disturbed health remain, is certain.

The author next passes in review several changes of structure to which the heart is liable, irrespective of inflammation affecting its lining or investing membranes. It may be the seat of each of the heterologous formations—carcinoma, tubercle, &c. Tubercular deposit is rare in that locality; Dr. L. has met with it in a few cases only. Analagous formations are more common, more especially the cartilaginous and osseus. Dr. L. regards these depositions as originally formed either beneath, or exterior to the membrane, both in the heart and arteries. The endocardium is much more frequently their seat than the pericardium, but specimens of the latter are occasionally met with. Laennec demonstrated that in the latter instance the deposit occurs between the fibrous and serous layers of the pericardium. These changes do not necessarily denote pre-existent inflammation, and from their imperceptible origin, and slow progress, it is difficult to refer them to any specific causes, either proximate or remote. They seem to be due in many cases to intemperate habits, a gouty diathesis, and to old age, singly or combined. With regard to diagnosis, the existence and seat of these lesions may be ascertained, but not their nature. The latter is only a matter of conjecture.

The muscular tissue of the heart may be the seat of acute inflammation terminating in the formation of pus. This, however, is extremely rare. Dr. Latham gives the report of one case which came under his own observation, and relates the history of the only other case of which he has any knowledge. The circumstances attending these cases afford nothing from which definite principles of diagnosis may be deduced.

Chronic inflammation affecting the muscular structure may lead to ulcerations, which may also originate from the pericardial, or endocardial surface, and penetrate inwards. Partial dilatations, sometimes called aneurisms, are among the consequences of changes exerted upon the muscular fibres. Under the latter circumstances rupture sometimes takes place, and this may be the first definite intimation that the heart is diseased.

Softening is another lesion, to which the attention of several observers has been of late years directed, especially as a complication of fever. Louis, Stokes and Hope, have pointed out this fact. According to these authorities, and the experience of Dr. Latham, a weak, irregular, intermitting pulse, attended with diminishde sounds and impulse in the præcordial region, often suffices to diagnosticate softening of the heart, which not infrequently proves the immediate cause of a fatal result. Softening is also found to occur in diseases characterised by an impoverished state of the blood, as scurvy, and chlorotic anæmia.

The predominance of fat in the heart occasions injury from its existing at the expense and detriment of the muscular fibres. It ends in dilatation of the cavities, and inducing extreme feebleness of the organ. The diagnosis can seldom reach beyond conjecture. Dr. L., states, however, "that when dilatation is ascertained by its appropriate signs, if valvular unsoundness, or its cause, be exclnded by the absence of endocardial murmur, and if a feeble, fluttering movement of the heart be felt at every part of the præcordial region, or beyond it; and if, moreover, the constitutional habit of the man be such as to accumulate fat in all other parts, then it may almost be taken for certain that fat is especially deposited upon the heart at the expense and detriment of its muscular substance."

Rupture of the heart is sometime due to the fatty degeneration. Dr. L., gives a case of that description which came under his observation.

The author now passes to the consideration of those affections which consist in alterations of *size* and *shape* and *bulk* and *capacity*, viz:—hypertrophy, atrophy, dilatation and contraction.

These affections are to be distinguished, after the language of the author, as, "unsoundness from disorganization." They do not denote any progressive disease properly so called, but follow, mechanically,from the lesions which prior disease has left. As regards the morbid anatomy, and pathological history generally, which appertains to these affections, Dr. L., does not advance any new facts; and our readers are presumed to be acquainted with the subject as fully, at least, as we should be able to present it in a brief analytical review. He does not consider under a distinct head their diagnosis. To their etiology he devotes considerable attention, and dwells particularly upon a cause not so fully noticed by other writers, viz:—"*shocks in which the heart suffers some hurt from violence.*" Hypertrophy with dilatation is especially apt to follow this class of causes. Dr. L., relates several cases, some from his own experience, and some from the recorded observations of others, in which the occurence of a heart affection was dated at the reception of some injury, generally from excessive muscular efforts; striking symptoms, such as sense of faintness, or sinking, palpitation, dyspnœa, &c., marking its occurrence. It is not easy to conclude what the nature of the injury is which the heart receives in these cases, no distinct inflammatory disease supervening. We quote his concluding observations on this topic:—

"But is it quite certain in these cases that the hypertrophy and dilatation really came from a material injury done to the heart at the time of the shock? In neither of them did the heart present the visible traces of any such injury as could be conceived to proceed immediately from violence. Still I do not know that any thing short of absolute disruption must necessarily leave the characteristic marks of itself ever afterwards. It is conceivable that the injury itself might not be of a permanent nature, and yet abide long enough to lay the foundation of permanent disorganization. Further it is possible that, in these same cases, causes might have been found in other parts of the body (for such it will presently appear there often really are) entitled to a share in producing what was found in the heart. Nevertheless the shock, that had been suffered in both cases, was a remarkable part of their clinical history. The patients themselves constantly ascribed to it the origin of all their malady. We cannot thererefore exclude it from our consideration, and may venture, without speculating further upon what cannot be proved, to regard it as in some manner powerfully conducive to the hypertrophy and dilatation of the heart, and to the fatal event."

The causes inducing this class of heart affections are often exterior to the heart, and may exist in organs remote, as well as near, and in the constitution at large. The operation of some of these causes is intelligible; others are, however, inexplicable.

Dilatation of the aorta leads to active or passive dilatation of the heart, to quote the language of the author, by "its own extraordinary efforts to overcome a virtual impediment to the circulation. Blood being immediately poured from it into a larger space than natural, requires from the heart an augmentation of its motive impulse." Most of the causes act directly, or indirectly, in the same way, i. e., they produce disorganization by occasioning some impediment either to the free passage of blood from the heart into the arteries, or to the circulation through organs which stand in close relation to the central organ.

Dr. L., remarks " it has never occured to me to meet with active or passive dilatation (hypertrophy with, and without dilatation) in a body otherwise perfectly sound. The concomitant diseases have not indeed had at all times a strictly accountable connection with it; yet, they have in a manner rendered its existence more intelligible." " But," he adds. "We must be cautious that we do not invert the real order of things. For the order of causation will be found to run as often from the heart to other organs, as from them to the heart." The relations of heart disease to affections of other organs, and to the condition of the general economy, constitute a subject of great pathological interest, and one upon which it behoves the practical physician to bestow close attention both in the abstract, and in the investigation ot individual cases.

The next three lectures of the work before us are devoted to the principles of treatment of the several diseases of the heart whose diagnosis, clinical history, causes, &c., have been considered. We shall present the general views advanced by the author with as much condensation as practicable.

In affections consisting of permanent valvular lesions, the treatment does not, of course, embrace any expectations of cure in the ordinary sense of the term. The sole object is to prevent the unsoundness from *becoming greater*. The evidence of valvular unsoundness, it is to be remarked, may be confined to the characteristic physical sign, the patient being not conscious of any disease whatever. An instance of this kind occured to us on the very day these remarks are penned. Other cases present themselves in which there is apparently perfect health to the observer, but the patient is aware that any exertions beyond a certain amount produce palpitations, dyspnœa, &c.

The details of treatment Dr. L., does not consider; they will be sufficiently obvious in every case in which the pathological condition of the heart and concomitant conditions are appreciated, the grand object being to moderate the operation of all existing causes and obviate new ones tending to produce impediment in the circulation, and excite the action of the organ. As Dr. L., remarks, "the first aim of the physician in such cases should always be to make the patient clearly to understand what his state is, and see the reasonableness of the advice that is given him. For his treatment, though it may proceed upon our suggestion, must be entirely carried on by himself. It must engage every hour of his life, and be allowed to interfere with all his habits, and conduct, and objects."

A certain amount of valvular unsoundness may continue for years without apparently any increase, nor with the effect of shortening life. We may therefore under favorable circumstances entertain a rational hope that this may be the case, but a positive prognosis should seldom be ventured upon.

Dr. Latham differs from Dr. Hope, and most writers respecting the curability of hypertrophy. According to Dr. Hope, most cases of that affection, before the age of forty years, may be radically cured, if uncomplicated with valvular disease, adhesion of pericardium, &c. On the other hand, Dr. Latham says, "in the whole course of my experience I never yet met with a single instance in which I was perfectly satisfied that it was cured." He is of opinion that those cases in which the affection was supposed to exist and a cure effected, were instances of "mock hypertrophy." Such instances he regards as very numerous, occurring in the prime of life from a "rich and redundant blood," in the dyspeptic and sensitive, &c. He consideres it unsafe to diagnosticate hypertrophy from mere forcible impulse of the heart, although it may be a very prominent and persistent symptom. We should quote his remarks at length on this topic if our space permitted, for they appear to us, in so far as may be allowed to judge from our limited observations, both just and practically important. We suspect that errors of diagnosis are very common with respect to hypertrophy. Not a few cases have fallen uuder our observation in which the error has been clearly declared by the result. The practical importance consists in the danger of depleting, &c., for the cure of that affection, when other, and different remedies would have been far better for the patient. We conceive it to be desirable to impress upon practitioners great caution in deciding upon hypertrophy when there are no evidences of valvular or pericardial lesions, more especially as, under these circumstances, it is extremely rare when compared with the numerous instances in which palpitation, &c., result from mere disturbance of the innervation of the organ. At the same time, it is not to be forgotten that the converse mistake would be fraught with disagreeable consequences.

Dr. Latham enjoins extreme care not to carry blood-letting too far in cases of hypertrophy. He says six or eight ounces should be the limit, by way of experiment, in any case with which there has been no previous acquaintance. Anœmia is to be carefully avoided. To quote the author's language, "it is an awful condition when it is added to hypertrophy in whatever way it arises."

Dr. L., in equally skeptical concerning the curability of atrophy and softening. With respect to the former he holds the following language:—

"As to attenuation of the heart, when it is a special disease of the organ itself, I cannot flatter myself that I ever cured it even in its simplest form. The complex form in which it commonly appears, may be safely pronounced incurable. The heart which is attenuated is commonly softened, and the heart which is attenuated and softened has commonly one, or both, of its ventricles dilated."

Softening, when it occurs in connection with fevers, chlorosis, &c., being dependent on conditions of the blood, and system at large, may be relieved by the treatment due to the latter; but when it occurs irrespective of these general conditions, it is hopeless.

"The patients are in the decline of life, or they have forestalled the season of old age by intemperate habits. Their nervous system may be shattered: their arteries may have undergone extensive changes of structure: their livers may be enlarged, their kidneys may be granulated. All of these forms of disease may be, and some of them are sure to be, conjoined with softening of the heart, before it comes to be known and to be treated."

The four lectures next following are devoted to the effects of heart disease as manifested in other organs, and the secondary affections resulting therefrom.

One of the most palpable of these effects is a distended, overloaded state of the veins, which is more apparent in the larger, superficial venous trunks, but more or less visible in all parts of the body.

Pulsation, or quivering, is sometimes observed, generally not extending beyond the jugulars, but occasionaly noticed even in the large veins of the hands and feet. This is attributable to a retrograde impulse communicated by the right ventricle through the tricuspid valves, but it does not necessarily indicate that these valves are unsound. Dr. Hope thought that this symptom might be produced without involving any regurgitation through the right auriculo-ventricular orifice. Dr. Latham adduces an anatomical fact, first pointed out by Hunter, to explain it, viz:—that these valves, in their healthy state, do not act so perfectly as barriers to regurgitation, as do the valves in the left ventricle. He regards this as an important provision, admitting of regurgitation when the heart is overloaded, and thus obviating accidents which might otherwise befall the lungs from an overplus of circulating fluid.

Dr. L., states that "dilatation of the right cavities is often the heart's sole or principal form of disorganization from first to last."

In this, his experience differs (if we do not mistake) from that of Dr. Hope, who regarded affections originating, or confined to the right side of the heart, as extremely rare.

From the accumulation of blood in the venous system arising from some impediment to the circulation through the central organ, follow *congestions*, *effusions*, *hemorrhages*, and *inflammations*.

The following propositions are submitted as expressing the sum of his remarks upon the limits which are to be considered as bounding our expectations from therapeutics in the several secondary forms of disorder just mentioned:—

"1st. That the fact of congestions, or effusions, or hemorrhages, or inflammations having their actuating cause in an unsound heart prohibits the possibility of their cure in *the highest sense*, and limits the expectations of medicine to their suspension, their abatement or their temporary removal.

2dly. That the form of unsoundness in the heart furnishes a measure of calculation, how much medicine will probably be able to effect in the individual case, whether it will go to suspend, or to abate, or even so far as to abolish them for a time.

3dly. That the presence or absence of coincident disease in other organs always modifies those expectations of relief by medicine, which the mere form of unsoundness in the heart itself would lead us to entertain.

4thly. That constitutional plethora and anæmia are grave contingencies when they are found in coincidence with an unsound heart, having an important bearing upon its consequences, and upon our modes of treating them, and upon our expectations of giving relief.

5thly. That, besides all these intrinsic conditions, the extrinsic accident of what may be the patient's circumstances in life throws a great weight into the balance for or against the probability of relief. The man, who, having an unsound heart, must traffic with his sinews, for his daily bread, has a poor chance of benefit from medicine.

6thly. That treatment has never more need of being favoured by opportunity than it has now.

Having thus formally stated the conditions intrinsic and extrinsic, which, being coincident with an unsound heart, give a character to its secondary diseases, and place them more or less within the reach of medical treatment, I shall venture to refer to them hereafter, without further explanation, as I may have occasion, when I come to speak expressly of these diseases and their management."

With respect to the location of the above mentioned secondary affections, the lungs are most liable to become their seat; next apoplectic coma, sometimes coming and going, no extravasation taking place, but, in other cases, true apoplexy resulting. Dr. L., has met with a case in which simply the violence of the heart's action upon the brain proved fatal without the lesions mentioned—the patient suffering from constant pain and vigilance, followed by mania and convulsions, and death occurring from exhaustiou of the nervous system.

The liver is frequently found greatly enlarged, from the long continued accumulation of blood in its vessels. But the affection which in the language of the author "chiefly declares the all-pervading and all-subduing power of the original malady of the heart," is *general dropsy*. In the pathological view of dropsy as a consequence of diseased heart, it is to be considered, that, in one sense, it is conservative—that is, the escape of serum is a measure of relief to the over distended vascular system. It is only the evils arising from the accumulation of the serum in the serous cavities, and cellular tissue which renders it a serious consequence. From this view of the subject our therapeutical inductions are to be derived. Here we quote the words of the author:—

"Seeing what nature is doing and must do, we can only go along with her, and seek to aid her in accomplishing her own purposes through other and less hazardous channels. Nature is seeking relief by directly evacuating the blood-vessels of their contents. We must try to gain for her the same relief by augmenting natural secretions, and so evacuating the blood-vessels through natural channels. The kidneys and the intestinal canal, and its subservient viscera, are the most eligible for the purpose.

The two concluding lectures of the work are occupied with the consideration of *angina pectoris*. The author relates some highly interesting and instructive cases of this obscure affection which have fallen under his observation, and the lectures contain much judicious practical advice with reference to its therapeutical and prophylactic management. We have, however, already extended this article so much farther than we had contemplated at the outset, that we must forego an analysis of, or extracts from this portion of the work.

In taking leave of the work (as a reviewer, for we do not bid adieu to it as a reader), we would repeat our acknowledgment of the gratification which we have derived from its perusal. As we have already said, it bears internal evidence of the earnest sincerity, and honest purpose of the author, not less than of his large and well improved opportunities for practical knowledge of the subjects of which he treats. These qualities furnish the chief reccomendations of works, which, like this, are designed to communicate important practical information for the medical practitioner. Mere literary merit is of secondary importance, although by no means unimportant even in treatises of that character. In the latter respect, this is without any ambitious pretensions, not to imply that it is particularly obnoxious to criticism. It would have been rendered more acceptable to the reader already somewhat conversant with the subject, if, instead of being in the form of lectures, which are apt to be discursive, it had been prepared with a more systematic, condensed, book-like arrangement, curtailed of many of its digressions and amplifications. But to the novitiate these are advantages rather than blemishes. As an introduction to the study of heart diseases, both for the student and practitioner, (for there are propably not a few of the latter who have given little or no attention to this department of practical medicine) these lectures are admirably adapted.

